# Research on Fault Diagnosis of Mechanical Bearings Based on Transfer Learning

**DOI:** 10.3390/s25247446

**Published:** 2025-12-07

**Authors:** Xinjian Gao, Yizhi Zhang, Enzhi Dong, Zhifeng You, Liang Wen, Zhonghua Cheng

**Affiliations:** Shijiazhuang Campus of Army Engineering University of PLA, Shijiazhuang 050003, China; 13912453267@163.com (X.G.); 15233119195@163.com (Y.Z.); ez_dong@aeu.edu.cn (E.D.); 19080118890@163.com (Z.Y.); lwenmark@163.com (L.W.)

**Keywords:** fault diagnosis, Transfer Learning (TL), Gradient Boosting Machine (GBM), Random Forest (RF), SHapley Additive Explanations (SHAP), mechanical bearing

## Abstract

Intelligent fault diagnosis is a set of methods for the health monitoring of mechanical bearings. To address the problem of insufficient applicability of diagnostic models due to differences in the domain distribution between laboratory data and actual working conditions, this study constructs a complete transfer learning diagnostic system. Firstly, the Hilbert transform technique was introduced to extract time-domain and frequency-domain features, as well as periodic correlations and other indicators; then, three models, i.e., transfer learning (TL), gradient boosting machine (GBM), and random forest (RF), were used to classify the data and compare their accuracy. It was found that TL had the highest accuracy in testing, with an F1 score of 0.9631. In the transfer task of the target domain samples, compared with the direct application of the source domain model with a classification accuracy of 70.3%, the transfer learning method achieved a classification accuracy of 97.6%, and the transfer gain increased by 27.3 percentage points, proving the superiority of the model constructed in this paper. Finally, SHapley Additive exPlanations (SHAP) was used to provide a detailed explanation of the transfer learning model, and the basis for model decision making was revealed through feature importance analysis.

## 1. Introduction

High-speed trains have become the mainstream mode of transportation for passenger transport due to their advantages of safety and efficiency [1]. As a key rotating component for the stable operation of high-speed trains, bearings are exposed to complex and harsh working conditions, such as high speed and alternating loads, for a long time [2]. They have the characteristics of high failure rate and being easy to damage, and they are also the main source of the equipment failures in the high-speed train transmission system. Once a bearing malfunctions, it can cause train delays and even lead to serious accidents, such as derailment, endangering life safety [3].

The current monitoring methods for high-speed railway bearings mainly rely on characteristic indicators constructed by expert experience or traditional signal processing techniques [4]. With the rapid development of rail transit systems towards high-density and strong coupling, existing methods are unable to meet the precise diagnostic requirements, in terms of diagnostic accuracy, generalization ability, and real-time performance, in complex operational scenarios [5]. In recent years, with the rapid development of big data and artificial intelligence technologies, data-driven intelligent fault diagnosis methods have received widespread attention in the field of train equipment operation and maintenance [6]. A deep learning model based on massive operational data can achieve higher precision fault recognition, stronger adaptability to working conditions, and more efficient real-time diagnostic capabilities [7]. However, in practical application scenarios, the raw vibration signals collected by sensors are easily affected by various sources, such as background noise and the interference source response due to the complex operating environments and changing working conditions [8]. This not only weakens the significance of fault features, but it also directly restricts the recognition accuracy of deep learning models [9]. Despite the advancements in conventional bearing condition monitoring techniques, several shortcomings persist, which motivate the present study. First, many data-driven models rely heavily on vast amounts of labeled historical data for training, which is often difficult or expensive to obtain in real-world industrial settings [10]. Second, these models frequently struggle with generalization, exhibiting performance degradation when confronted with unforeseen fault types or operating conditions that are not present in the training dataset [11]. Furthermore, traditional vibration analysis methods can be sensitive to noise and changes in machine load or speed, necessitating robust feature extraction and domain adaptation techniques. In this context, transfer learning technology provides a good approach to solving the above-mentioned problems.

**Figure 1:**
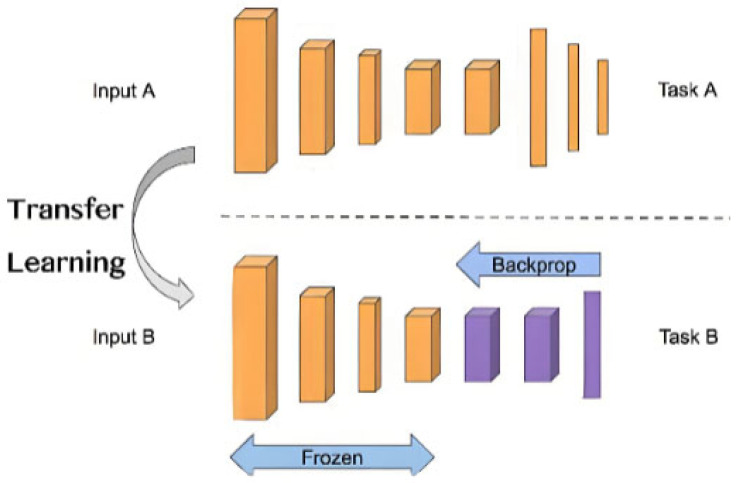
The process of transfer learning.

Transfer learning technology, where the core idea is to transfer the knowledge learned in one task or domain to another related but different task or domain (in order to improve the model performance of the target task), is an emerging machine learning method [12]. Therefore, by analyzing the mechanism of bearing faults on existing test bench data, extracting representative bearing fault features to construct a source domain model, and combining transfer learning techniques to transfer diagnostic knowledge to actual operational train data, the problem of sample imbalance can be effectively alleviated [13]. (The process of transfer learning is shown in Figure 1.)

Transfer learning is a machine learning method that utilizes the knowledge learned in the source domain (source task) to improve the learning performance of the target domain (target task), thus reducing the amount of training data required in the target domain and improving learning efficiency [14]. Transfer learning includes two important concepts.

(1) Domain: The subject of model learning, mainly composed of data and its probability distribution [15]. This question covers two domains, namely the bearing test bench data and its distribution (source domain) and the actual operational train data and its distribution (target domain).
(2) Task: The task is the specific goal that the model needs to achieve in the domain, and its mathematical essence is to achieve the mapping relationship from input space to label space by constructing a prediction function. Among them, the input space represents the set of all possible input samples, and the label space represents the set of output values (labels) that the model needs to predict [16]. In this question, the specific tasks include the classification task of the bearing test bench data (source task) and the classification task of the actual operating train data (target task).

The pseudocode for transfer learning is shown in Algorithm 1.
**Algorithm 1.** Pseudocode for running the algorithm.
1: import torch2: import torch.nn as nn3: import torch.optim as optim4: **from** torchvision import models, datasets, transforms5:      Loading the pre-trained model6: **model** = models.__dict__[PRETRAINED_MODEL](pretrained=True)7:      Freeze parameters (feature extraction strategy)8: **if** STRATEGY == ‘feature_extraction’:9:      **for** param in model.parameters():10:    param.requires_grad = False 11: **Replace** the last fully connected layer (classification head)12:    Get the input feature count of the original full connection layer13:    num_ftrs = model.fc.in_features14: **Replace** with a new layer that matches the target category count15:    model.fc = nn.Linear(num_ftrs, NUM_CLASSES)16: **Loss function and optimizer**17:    criterion = nn.CrossEntropyLoss()18:    Optimizer: Only train parameters that require gradients19: **if** STRATEGY == ‘feature_extraction’:20:    Only train the newly added FC layer21:    optimizer = optim.Adam(model.fc.parameters(), lr=LEARNING_RATE)22: **elif** STRATEGY == ‘finetune’:23:    Train all parameters, but use a smaller learning rate for the pre-training layer24:    optimizer = optim.Adam(model.parameters(), lr=LEARNING_RATE)25: **Training cycle**26:    device = torch.device(“cuda:0” if torch.cuda.is_available() else “cpu”)27:    model = model.to(device)28:    for epoch in range(EPOCHS):29: **Verification phase**30:    Save model31:    torch.save(model.state_dict(), ‘transfer_learning_model.pth’)32:    print(“Completed!”)

The working environment of train bearings is often harsh. During long-term operation, bearing components may be damaged due to various factors, such as overheating, poor lubrication, corrosion, etc., which can affect the normal operation of the train [17]. Bearing fault diagnosis refers to the technology of using data mining, signal processing, and machine learning techniques to determine whether a bearing has a fault by monitoring various signals (with vibration acceleration signals as the main monitoring data) during the operation of the bearing. Essentially, machine learning-based fault diagnosis problems belong to pattern recognition problems extracting fault features through signal processing and other techniques, which is then followed by using machine learning models to achieve accurate classification (a common implementation path in this field). Its core goal is to achieve early warning and accurate diagnosis of faults, thereby avoiding sudden equipment failures and providing reliable guarantees for the safe operation of trains.

**Figure 2:**
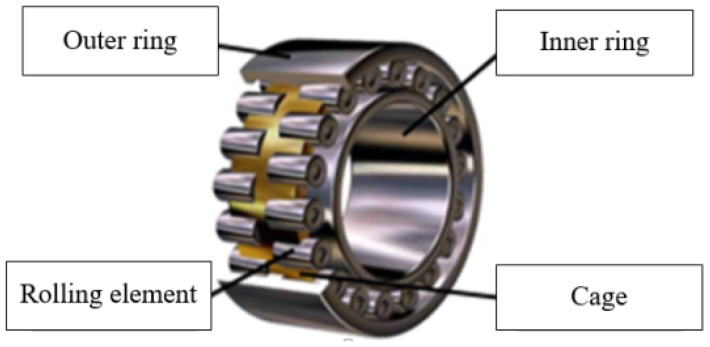
Schematic diagram of the bearing structure.

The bearing structure mainly includes four parts: the inner ring, outer ring, rolling elements, and cage. Typical faults often occur in the following three core load-bearing components: the inner ring, outer ring, and rolling elements [18]. When a local defect occurs in a bearing, the rolling elements will generate a sudden impact pulse at the moment of contact and will then pass through the defect point [19]. During the periodic operation of the bearing, this pulse force will continue to act, forming periodic impact components. The structural composition of the bearing is shown in Figure 2.

Due to differences in structural function and stress characteristics, the vibration signals of different parts exhibit different characteristics when faults occur [20].

## 2. Problem Description

Bearings are the core support components of a high-speed train transmission system, operating under complex dynamic loads for extended periods. They are prone to failures at the inner ring, outer ring, and rolling elements due to factors such as fatigue and wear, posing a threat to train safety. The key to achieving precise diagnosis lies in effectively extracting fault characteristics [21]. Given the diverse physical mechanisms of different faults, their vibration signals all manifest as periodic impacts related to the geometric parameters of the bearing, rotational speed, and fault type. Therefore, in-depth analysis of fault mechanisms and the establishment of mathematical models linking characteristic frequencies to bearing parameters serve as the essential theoretical foundation for feature extraction and diagnosis [22]. The time-domain characteristics of bearing faults are directly correlated with their physical mechanisms: regular impacts in outer ring faults stem from the stationary nature of defect locations; amplitude fluctuations in inner ring faults result from the modulation effect of defect points rotating with the shaft; while the complex characteristics of rolling element faults arise from the combined action of their spin and orbit motion. The prominence of these features increases with fault degradation, where time-domain analysis not only reveals the evolution patterns of faults, but it also lays a solid theoretical and practical foundation for subsequent modeling [23].

The characteristic frequency of inner race fault (BPFI) is generated by the periodic impact between the rolling elements and the rotational defect, and its expression must account for the relative rotational speed between the inner race and the rolling elements. $f_{r}$ is the bearing rotation frequency; *n* is the inner ring speed of the bearing (unit: rpm), $f_{r}$ = *n*/60; *d* is the rolling element diameter; *D* is the bearing pitch diameter (usually estimated by [inner diameter + outer diameter]/2); and *N_d_* is the number of rolling elements. Under normal operating conditions, where the outer race remains stationary while the inner race rotates with the shaft, we have the following:

(1) $$BPFI=\frac{N_{d}}{2}\cdot \left(1+\frac{f_{r}\cdot d}{D}\right).$$

The outer race fault characteristic frequency (BPFO) originates from the periodic impacts of rolling elements sequentially striking fixed defect points. If so, then the following applies:

(2) $$BPFO=\frac{N_{d}}{2}\cdot \left(1-\frac{f_{r}\cdot d}{D}\right).$$

This formula indicates that, for each revolution of the inner ring defect, the number of impacts of the rolling elements on it is greater than that of the outer ring defect—so *f_BPFI_* > *f_BPFO_*—and the frequency spectrum is modulated with the axis rotation frequency, presenting a sideband structure, which can be used for the identification and verification of inner ring faults.

The characteristic frequency of rolling element failure (BSF) originates from the collision between the defect and the inner and outer raceways once per rotation of the rolling element, and this is accompanied by the revolution effect of the rolling element. The calculation formula is as follows:

(3) $$BSF=\frac{D}{d}\cdot \left[1-{\left(\frac{f_{r}\cdot d}{D}\right)}^{2}\right].$$

This frequency reflects the double impact period caused by the rotation of the rolling element, usually lower than the fault frequency of the outer and inner rings, and it may be accompanied by frequency modulation sidebands of the cage in the spectrum, which is an important basis for identifying local peeling of the rolling element.

For a discrete vibration signal sequence {x1, x2,…, xN} of length N, the commonly used time-domain statistical characteristics are defined as follows. Among them, the mean is used to measure the average level of the signal:

(4) $$u=\frac{1}{N}\sum_{i=1}^{N}x_{i}.$$

The standard deviation reflects the degree of signal fluctuation:

(5) $$\sigma =\sqrt{\frac{1}{N}{\sum_{i=1}^{N}\left(x_{i}-u\right)}^{2}}.$$

Root mean square represents the energy level of the signal:

(6) $$RMS=\sqrt{\frac{1}{N}{\sum_{i=1}^{N}x_{i}}^{2}}.$$

Kurtosis represents the impulse characteristics of a signal:

(7) $$k=\frac{\frac{1}{N}{\sum_{i=1}^{N}\left(x_{i}-u\right)}^{4}}{\sigma^{4}}.$$

Skewness can quantify the symmetry of the distribution shape of vibration signals. In fault diagnosis, it is often used to identify asymmetric impact behavior:

(8) $$S=\frac{\frac{1}{N}{\sum_{i=1}^{N}\left(x_{i}-u\right)}^{3}}{\sigma^{3}}.$$

The margin factor is an indicator that is sensitive to changes in impact amplitude and is commonly used for early fault diagnosis:

(9) $$MF=\frac{x_{peak}}{{\left(\frac{1}{N}\sum_{i=1}^{N}\sqrt{\left|x_{i}\right|}\right)}^{2}}.$$

The pulse factor can evaluate the significance of the instantaneous impulse peak in the signal relative to the average level. The larger the value, the more prominent the impact component:

(10) $$IF=\frac{x_{peak}}{\frac{1}{N}\sum_{i=1}^{N}\sqrt{\left|x_{i}\right|}}.$$

These time-domain features form a multi-dimensional analysis framework: the mean, standard deviation, and RMS reflect the overall statistical characteristics and energy level of the signal; the kurtosis and skewness characterize the morphological characteristics of its probability distribution; and the margin factor and pulse factor have become effective tools for detecting early bearing failures due to their high sensitivity to sudden impacts.

The extraction of frequency-domain features aims to capture the frequency structure information of the signal. Therefore, first, the frequency spectrum of the signal is obtained through fast Fourier transform (FFT), and the analysis focuses on quantifying the fault characteristic frequency and the amplitude corresponding to each harmonic order.

(11) $$X(f)=\int_{-\infty }^{+\infty }x(t)e^{-j2\pi ft}dt.$$

To effectively demodulate and enhance the periodic impulse components in vibration signals, this study introduces the envelope analysis technique based on Hilbert transform. This method extracts the envelope by constructing the analytical signal *Z*(t) = *X*(t) + *jH* [*X*(t)] of the signal *X*(t) and calculating its modulus. Its mathematical definition is as follows:

(12) $$X_{env}(t)=\left|Z(t)\right|=\left|X(t)+jH\left[X(t)\right]\right|.$$

**Figure 3:**
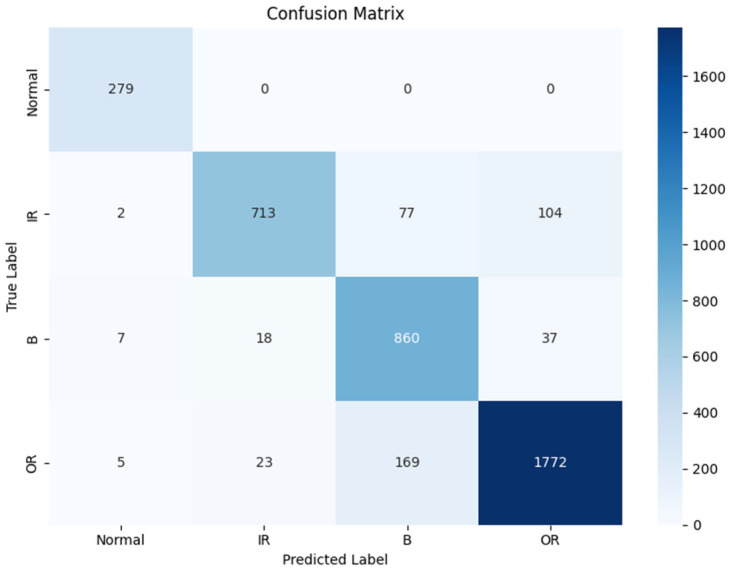
Confusion matrix of the original data.

Among them, *H* [·] represents the Hilbert transform operator. Directly using the raw data, 1000 data points were cut to achieve enhancement, and these were then placed into a residual convolution model with an attention mechanism. The resulting confusion matrix is shown in Figure 3.

Segmenting the signal into fixed-length epochs of 1000 points standardizes the input dimensions for wavelet transform. The 1000-point segmentation is predicated on the assumption of quasi-stationarity within short time frames. This approach also helps in isolating and mitigating the impact of transient, high-amplitude noise that might be present in other parts of the signal.

**Figure 4:**
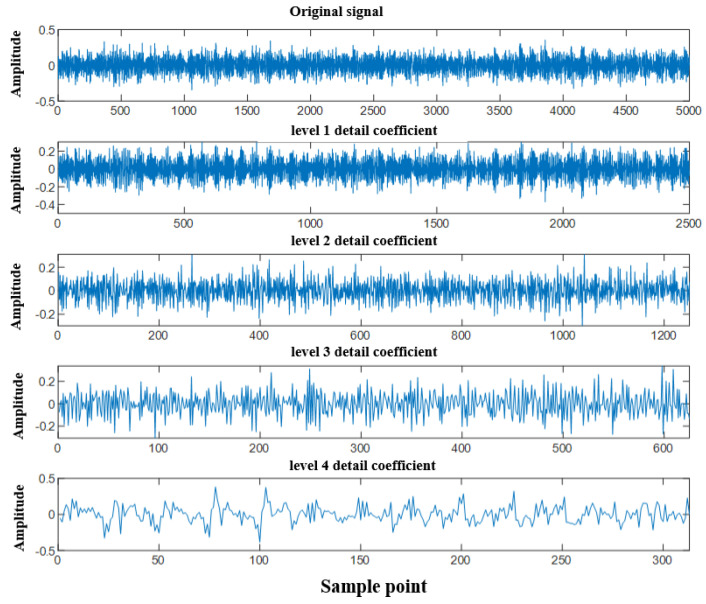
The coefficient values of each layer.

After cutting the signal into 1000 points, perform wavelet transform on the cut signal to obtain multi-scale coefficients. Due to the fact that wavelet coefficients from different layers can, respectively, characterize the high-frequency and low-frequency characteristics of signals (and complement each other), the first, second, third, and fourth layers of wavelet coefficients are placed in a multi branch neural network model, and the residual attention blocks are used to extract features. Finally, attention mechanisms are used for multi branch feature fusion (the coefficient values of each layer are shown in Figure 4).

**Figure 5:**
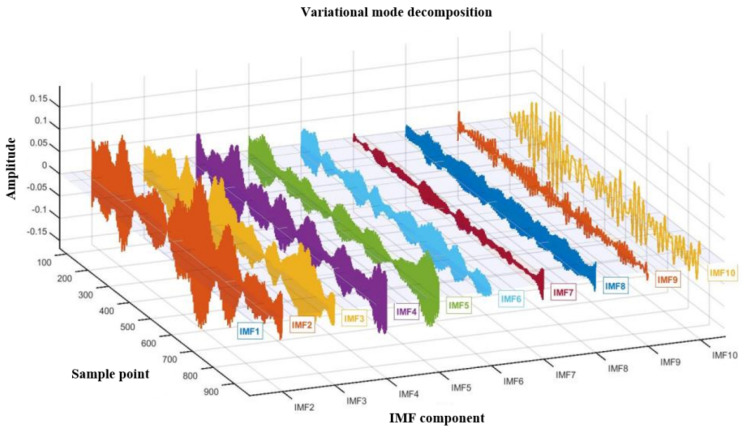
Variational mode decomposition after feature extraction.

Variational mode decomposition (VMD), where the core idea is to iteratively search for the optimal solution of the variational model and adaptively decompose the original signal into a series of finite bandwidth intrinsic mode functions (IMFs) with specific center frequencies, is a completely non recursive signal decomposition method [24]. VMD technology aims to decompose complex signals into several modal components. This process constructs and solves variational problems to ensure that each mode is closely centered around its center frequency in the spectrum, effectively overcoming the phenomenon of mode aliasing. Unlike recursive decomposition methods, such as Empirical Mode Decomposition (EMD), VMD transforms the signal decomposition problem into an optimization problem within a variational framework. By constraining the bandwidth and center frequency of each mode, VMD achieves a more robust and mathematical decomposition. The variational mode decomposition after feature extraction is shown in Figure 5. To statistically validate the effectiveness of decomposition, this study conducted *t*-tests comparing each IMF component with the original signal residuals. The results showed no significant correlation between modalities and residual sequences (*p* > 0.05), demonstrating that VMD successfully extracted the primary components without retaining significant structural information, thereby confirming the completeness of its decomposition. Additionally, variance analysis revealed significant differences in the IMF center frequencies across operational conditions (*p* < 0.01), quantitatively validating the effectiveness of VMD decomposition for subsequent feature analysis and state recognition.

**Figure 6:**
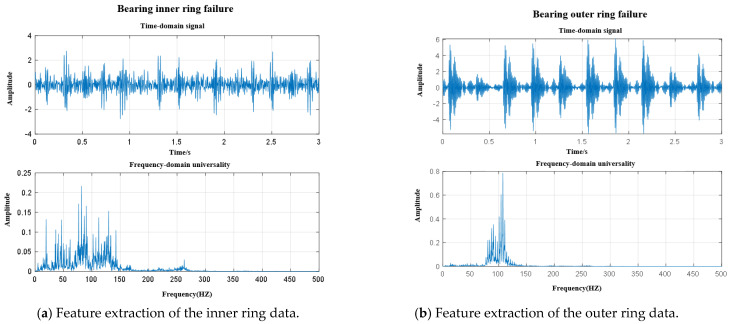

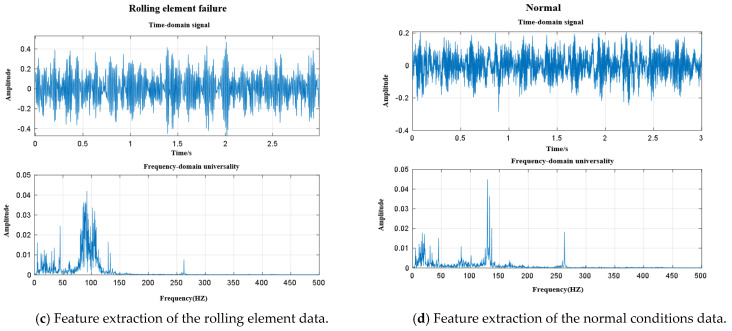
Data feature extraction under different operating conditions.

The analysis results of the vibration signal data of the outer ring (OR), inner ring (IR), and rolling element (RE) faults and normal state (N) of the train rolling bearings after feature extraction are shown in Figure 6.

The evaluation indicators for the raw data and feature extracted data are shown in Table 1.

The evaluation indicators of the data after feature extraction were significantly better than those of the original data before feature extraction, proving the effectiveness of feature extraction. The importance ranking of each feature is shown in Table 2.

## 3. Model Construction

The recognition of bearing defects is limited by high-dimensional nonlinear signals and multimodal faults, and a single learner is prone to underfitting [25]. Multi model fusion utilizes the error compensation between heterogeneous base learners to simultaneously compress variance and bias (tree methods mine feature interactions, linear equations provide interpretable weights, and distance measures capture local impulse beats), thereby achieving synchronous improvement in accuracy and robustness in small sample and strong noise scenarios.

Random Forest Classifier (RF): Random forest uses “multi tree voting” to reduce the risk of misdiagnosis [26]. First, bootstrap resampling is performed on the training set *D* to generate several sub samples. Then, when each tree node splits, a feature subset is randomly selected to ensure inter tree diversity and suppress overfitting. Finally, taking the mode vote as the output significantly improves the diagnostic stability in strong noise environments. Given training set *D*, we have the following:

(13) $$D=\left\{\left(x_{1},y_{1}),(x_{2},y_{2}),\ldots ,(x_{N},y_{N}\right)\right\},x_{i}\in {\mathbb{R}}^{d},y_{i}\in \left\{1,2,\ldots K\right\}.$$

In a random forest, M decision trees are trained in parallel. Given a sample x, each tree $h_{m}(x)$ outputs a category label, and the final prediction result is determined by the statistical patterns of these outputs:

(14) $$H(x)=\arg\max\sum_{m=1}^{M}I(h_{m}(x)=k),\;k\in \left\{1,2,\ldots K\right\}.$$

Among them, I(⋅) is the indicator function, which is defined as follows: when the condition $h_{m}(x)=k$ is met, the function value is 1; otherwise, it is 0.

**Figure 7:**
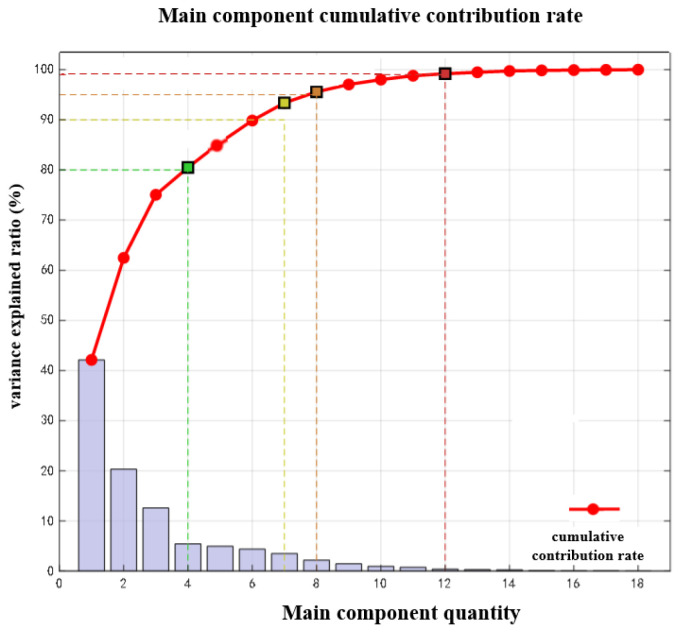
The cumulative contribution rate of the principal components.

The main components of the fault data are shown in Figure 7.

The gradient boosting machine (GBM) adopts an additive modeling strategy: each decision tree grown sequentially no longer votes independently but rather fits the negative gradient of the previous round’s residuals, gradually correcting the predicted values with “tree + learning rate” and finally outputting the cumulative result, thus obtaining tighter classification boundaries and higher generalization performance than random forests in noisy environments [27]. Given the training dataset, we have the following:

(15) $$D={\left\{\left(Y,T_{i}\right)\right\}}_{i=1}^{N},x_{i}\in {\mathbb{R}}^{d},T_{i}\in \left\{1,2,\ldots K\right\}.$$

After iterative training, the prediction model of GBM integrates M weak learners (decision trees), and the final prediction result is a linear superposition of the outputs of these trees.

(16) $$\overset{\wedge }{T_{i}}=\sum_{m=1}^{M}W_{m}(Y_{i}),\;W_{m}\in F.$$

The mapping function for the m-th regression tree is $W_{m}(x)$, where F represents the function space composed of all regression trees. The objective function of GBM consists of a loss term and a regularization term added together:

(17) $$J(\phi )=\sum_{i=1}^{N}G(T_{i},\overset{\wedge }{T_{i}})+\sum_{m=1}^{M}R(W_{m}).$$

The regularization term $R(W_{m})=\gamma T+0.5\lambda {\left\|W\right\|}^{2}$ constrains the model complexity, where W is the weight of leaf nodes, T is the number of leaf nodes, and γ and λ control the penalty strength to prevent overfitting. The loss function $G(T_{i},\overset{\wedge }{T_{i}})$ is used to quantify the deviation between the current prediction and the true label, and Softmax regression can map the network output vector to a probabilistic simplex.

(18) $$P_{ik}=\frac{\exp({\overset{\wedge }{T}}_{ik})}{\sum_{j=1}^{k}\exp({\overset{\wedge }{T}}_{ij})},{\overset{\wedge }{T}}_{ik}=\sum_{m=1}^{M}W_{m}{\left(Y_{i}\right)}_{k},$$

where i and k represent the sample and category, and the model outputs a normalized probability of $P_{ik}$.

Transfer learning can overcome the data distribution shift between the source domain (laboratory bench) and the target domain (on-site operation). The controllable, low-noise, and fixed load characteristics of the test bench data are in stark contrast to the complex and ever-changing on-site environment, leading to a decline in the performance of the direct transfer model. This domain difference is not only reflected in the signal amplitude, but it also changes the frequency domain and time-frequency distribution patterns of the fault characteristics [28]. Therefore, transfer learning breaks through the limitations of traditional supervised learning by specifically handling the distribution bias between labeled source domains and unlabeled target domains (i.e., the edge distribution differences caused by the collection environment, as well as the conditional distribution differences caused by changes in the fault response with operating conditions).

The core task of transfer learning in bearing fault diagnosis is to compensate for the data drift between the two “laboratory field” scenarios. The experimental platform signal is controlled, clean, and has a single operating condition, while the service data is mixed with variable loads, coupled noise, and external environmental disturbances [29]. This difference not only scales the amplitude, but it also reshapes the energy distribution of fault characteristics in the frequency band and time-frequency plane. To this end, the transfer strategy incorporates the “edge drift caused by different collection conditions” and the “condition drift triggered by changes in operating conditions” into the adaptation framework, freeing the model from the strict dependence of traditional supervised learning on samples with the same distribution [30].

Set the target domain $S_{t}=\{k_{j}^{t}{\}}_{j=1}^{n_{t}}$, source domain $S_{h}=\{(k_{i}^{h},l_{i}^{h}){\}}_{i=1}^{n_{h}}$, l is the label, and K is the characteristic of vibration signal. This study used the Maximum Mean Discrepancy (MMD) to measure and reduce the inconsistency of the feature distributions between two domains. The definition of this statistic is as follows:

(19) $${\mathrm{MMD}}^{2}(S_{h},S_{t})={\left\|\frac{1}{n_{h}}\sum_{i=1}^{n_{h}}\;\phi (k_{i}^{h})-\frac{1}{n_{t}}\sum_{j=1}^{n_{t}}\;\phi (k_{j}^{t})\right\|}_{H}^{2}.$$

The supervised loss function on the source domain data is retained, $W_{ce}=-\sum llog\overset{ˆ}{l}$. Measure distribution differences by calculating the embedding distance of features in the reproducing kernel Hilbert space (RKHS; the norm is $\|\cdot {\|}_{H}$). Minimizing this joint objective can effectively align the two domain distributions and promote the cross domain invariance of features.

(20) $$W=W_{ce}+\lambda {\mathrm{MMD}}^{2}(S_{h},S_{t}),$$

where λ is a hyperparameter used to balance two losses. This design aims to learn a feature representation that is equally applicable to the target domain while ensuring that the classification performance of the source domain is not compromised, ultimately building a robust feature foundation for transfer diagnosis. The primary objective of this study is to extract essential fault features that are insensitive to domain labels in order to overcome the model performance degradation caused by differences in the sampling rate, operating spectrum, and noise base between the test bench and train data. The specific method is to construct a shared embedding space: first, fuse the time-domain statistical indicators, frequency-domain fault characteristic peaks, and short-term energy spectra to form a high-dimensional mixed feature vector [31]; subsequently, through normalization and distribution alignment techniques, the difference in marginal distribution between the source domain and the target domain is reduced, ultimately making it impossible for the classifier to distinguish the source of the samples in this common space, thus focusing on the discrimination of the fault itself [32].

The target domain is often difficult to effectively train in machine learning due to scarce annotations and imbalanced samples. Transfer learning solves this problem by utilizing the rich knowledge and data in the source domain. It transfers the knowledge learned in the source domain to the target domain, which not only reduces its dependence on data volume and accelerates the learning process, but also significantly enhances the generalization performance of the model. To solve the diagnostic problem caused by insufficient labeling and real samples in the target domain (actual train data), this study used transfer learning techniques, such as feature mapping and model fine tuning, to transfer diagnostic knowledge from the source domain (test bench data) to construct a robust model that can effectively classify unlabeled data in the target domain.

## 4. Experimental Verification

The focus of this study is to achieve effective knowledge transfer. Specifically, the aim is to transfer diagnostic knowledge with rich labels and clear physical meanings from the source domain (bearing test bench) to the target domain (train operation data) with fuzzy fault types, significant noise, and scarce samples. Therefore, the technical key lies in designing a migration mechanism that can overcome the inter domain differences mentioned above, ultimately enabling the model to have the ability to identify unknown faults in the target domain.

Gradually remove or replace each module and observe performance changes. ① Feature extraction module: responsible for extracting features with generalization ability from the source or target domain. ② Distribution alignment module: improves cross domain generalization ability by reducing the distribution difference between the source domain and the target domain. ③ Model fine tuning module: Fine tunes the pre trained model on the target domain data to adapt to the target task.

(1) Source dataset (bearing test bench vibration data with minor noise)

The test bench’s main structure consists of an electric motor, comprising three primary components: the drive end, fan end, and base. Acceleration sensors installed on the motor housing, fan end, and base collect operational data. Both the drive end and fan end are equipped with test bearings. These bearings are designed to generate single-point failures through intentional damage (resulting in isolated defects at specific locations), eliminating compound failures. Each data set only records one bearing failure occurrence.

(2) Platform bearing information

The bearing to be tested supports the motor shaft;

The drive-side bearing is SKF6205, with sampling frequencies of 12 KHz and 48 KHz.

The fan’s bearing is SKF6203, with a sampling frequency of 12 KHz.

The bearing size parameters are shown in Table 3.

(3) Data formats and variable names

The data files are in MATLAB’s .mat format, with each file containing different types of data. Variable names are defined as follows: DE (drive end accelerometer data), FE (fan end accelerometer data), BA (base accelerometer data), time (time series data), and RPM (revolutions per minute during testing, divided by 60 to obtain rotational frequency).

(4) Bearing operating status category

The dataset contains four operational states for bearings: Outer Ring Fault (OR), Inner Ring Fault (IR), Rolling Element Fault (B), and Normal Operation (N). Specifically, there are 77 OR fault samples, 40 IR fault samples, 40 B fault samples, and 4 N samples. OR faults exhibit three distinct dimensional variations: 0.007, 0.014, and 0.021 inches. Both IR faults and B faults demonstrate four dimensional variations: 0.007, 0.014, 0.021, and 0.028 inches. Given the fixed positional nature of OR faults (unaffected by bearing rotation), vibration signals are comprehensively captured at three orthogonal angles: 3 o’clock (Orthogonal), 6 o’clock (Centered), and 12 o’clock (Opposite). The load conditions are categorized as 0, 1, 2, and 3 horsepower.

(5) Target Domain Dataset (Train Bearing Fault Dataset)

This dataset contains vibration signal data from rolling bearings (outer ring, OR; inner ring, IR; rolling elements, B) under fault and normal conditions (N). The data is collected at 8-s intervals with a sampling frequency of 32 kHz, at approximately 90 km/h train speed (bearing rotation speed: 600 rpm). The data files are named with English letters A to P, and the operational status of each dataset remains unspecified.

This study addresses the issue of inter domain differences through feature mapping and model fine tuning. Feature mapping aims to project heterogeneous data onto a shared feature space to align distributions; model fine tuning refers to retraining the pre trained model in the source domain to adapt to the target domain. This path achieves effective transfer of source domain knowledge to the target domain, balancing training efficiency and the model’s ability to discriminate new labels.

Specifically, a Convolutional Neural Network (CNN) is used as the foundational architecture. Its advantage lies in the universality of the feature extractor learned on the source domain, and through fine tuning, the model can quickly focus on the unique features of the target domain, achieving efficient domain adaptation. The traditional feature transfer method overcomes the distribution shift between the source domain and the target domain through a shared feature space. The key is to use a mapping function ϕ to transform the two domain data in order to achieve distribution alignment and to ensure effective model transfer.

(21) $$x_{s}=\phi (x_{s}^{i}),x_{t}=\phi (x_{t}^{i}).$$

The function ϕ defines the mapping relationship from the original domain to the common feature space X. Transfer the pre trained CNN model from the source domain $f_{S}(x_{s})$ to the target domain and achieve adaptation by fine tuning its parameters (weight W and bias b).

(22) $$y_{t}=f_{T}(x_{t})=Wf_{S}(x_{t})+b.$$

The fine-tuning process refers to targeted optimization of W and b to make the output yt more in line with the true situation of the target domain. This strategy fully utilizes the annotation information of the source domain, effectively alleviating the constraint of insufficient labeled data in the target domain, thereby obtaining accurate fault diagnosis results. Transfer learning aims to improve the prediction accuracy of the target domain through source domain knowledge, which is often formalized as an optimization problem.

(23) $$L(D_{T},\theta )=\frac{1}{N_{t}}\sum_{i=1}^{N_{t}}\;L(y_{t}^{i},f_{T}(x_{t}^{i};\theta )).$$

To reduce the impact of inter domain distribution differences on model performance, the loss function in transfer learning usually consists of two parts: a loss term used to ensure the performance of the source domain task, and a loss term used to promote adaptation to the target domain.

(24) $$L_{total}=L_{source}(D_{S},\theta )+\lambda L_{target}(D_{T},\theta ).$$

$L_{source}(D_{S},\theta )$ can be further expressed as follows:

(25) $$L_{source}(D_{S},\theta )=\frac{1}{N_{s}}\sum_{i=1}^{N_{s}}\;L(y_{s}^{i},f_{S}(x_{s}^{i};\theta )).$$

Feature mapping is a core technique used in the field of transfer learning to reduce the distribution differences between domains. The core idea is to transform the data from the source and target domains into a shared feature space X in order to achieve distribution alignment within that space.

(26) $$x_{s}=\phi (x_{s}^{i}),x_{t}=\phi (x_{t}^{i}).$$

Among them, the function ϕ defines the mapping from the original domain to the common feature space X. In order to pursue better distribution matching, an adversarial training mechanism is often used. For example, the feature mapping process can be embedded into the framework of Generative Adversarial Networks (GANs) for optimization.

(27) $$L_{adv}=E_{x_{t},x_{s}}[D(\phi (x_{s}))-D(\phi (x_{t}))],$$

**Figure 8:**
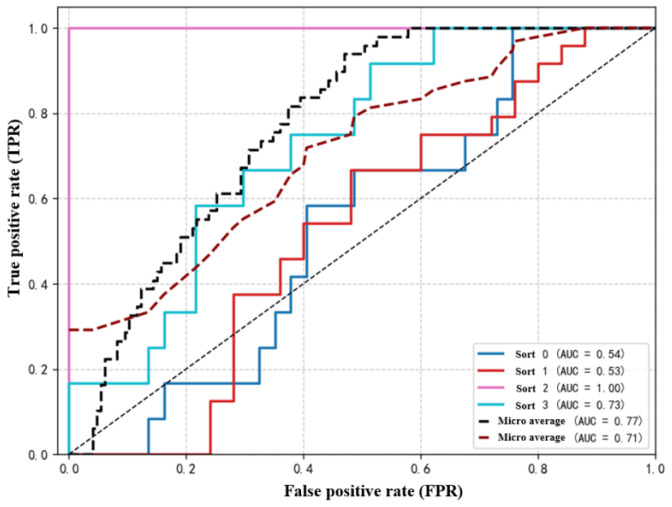
The false positive rate of the training data.

The essence of discriminator *D* is a classifier used to determine the domain to which a feature belongs. The ultimate goal of adversarial training is to train the feature mapping module to produce features that can confuse the discriminator until it is unable to make accurate judgments, thereby achieving the conditions required for transfer learning. The false positive rate of the training data is shown in Figure 8.

Fine tuning, which refers to the process of applying a pre trained model on the source domain to the target domain and conducting targeted optimization, is a key step in transfer learning. The usual approach is to transfer the network structure and weights of the pre trained model as initial values and then continue only training on the target domain data to optimize its classification loss. The objective function of this process can be defined as follows:

(28) $$L_{t}=L_{s}(D_{S},\theta_{pre})+L_{target}(D_{T},\theta_{fin}).$$

The parameters of the source domain pre trained model are $\theta_{pre}$, and the parameters of the target domain model after fine tuning are $\theta_{fin}$. The core problem that sample-based transfer learning aims to solve is the difference in the data distribution between the target domain and the source domain. This method achieves this goal by weighting the importance of source domain samples, that is, increasing the weight of samples with high similarity to the target domain so that they can play a greater role in training.

(29) $$L_{weight}=\frac{1}{N}\sum_{i=1}^{N}\;R_{i}L(y^{i},f(x^{i})).$$

The mechanism for assigning weight Ri to sample *i* is determined by the similarity between the overall distribution of the source domain samples and target domain samples. The key mechanism for achieving cross domain knowledge transfer is to transfer the data distribution knowledge represented by the source domain to the target domain in order to optimize its learning performance under limited supervised information. Due to the difference in the feature dimensions between the two domains, direct transfer is not feasible. In view of this, first, feature alignment is performed through semantic mapping to construct a common feature space with a same dimension in order to establish the correspondence between features.

To standardize the distribution between domains, Z-score normalization is applied to feature data.

(30) $$X_{normal}=\frac{X-u}{\sigma }.$$

**Figure 9:**
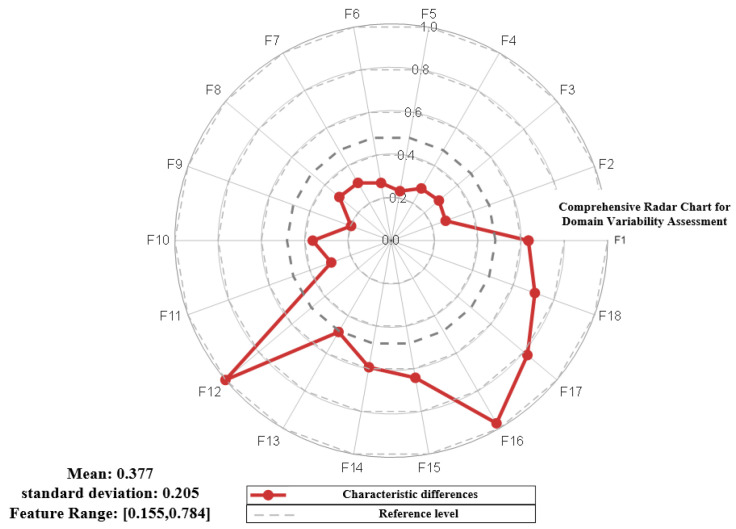
Radar chart of the data evaluation after implementing transfer learning.

Using distribution distance as a metric to quantitatively measure the difference between the source domain and the target domain, its definition is as follows:

(31) $$D_{dist}={\left\|V_{S}-V_{T}\right\|}_{2}+{\left\|O_{S}-O_{T}\right\|}_{2},$$

where $V_{T}$, $O_{T}$, $V_{S}$, and $O_{S}$ are statistical parameters for the target domain and source domain, respectively. The radar chart for evaluating the data differences after implementing transfer learning is shown in Figure 9.

In evaluating the transfer effect, this study used transfer gain to quantify the effectiveness of transfer learning. The calculation formula for this indicator is as follows:

(32) TG=Acc_transfer−Acc_direct.

**Figure 10:**
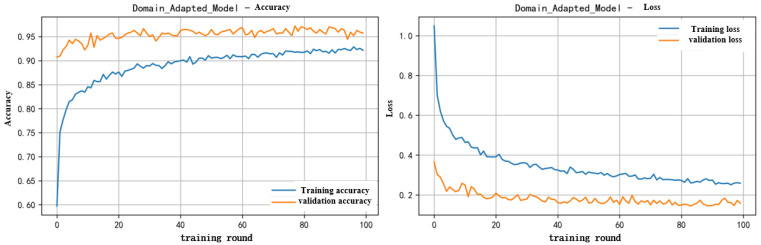
Mixed training accuracy and loss graph.

Among them, Acc_transfer represents the accuracy of the model on the target domain after using transfer learning, and Acc_transfer represents the accuracy obtained by directly applying the source domain model to the target domain. Firstly, perform coronal alignment to align the target domain with the source domain. Use the model trained from the source domain to predict the target domain as pseudo labels, and then discard the samples with low confidence. Then, the target domain data with pseudo labels and the source domain data are mixed and trained using the source domain model. The accuracy and loss of the mixed training data are shown in Figure 10.

**Figure 11:**
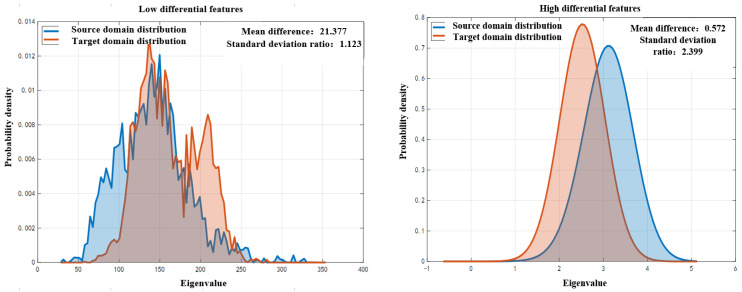
Comparison of the low- and high-differential features between the source and target domains.

A comparison of the low and high difference features between the source and target domains is shown in Figure 11.

As shown in Figure 11, there are domain differences between the source domain and the target domain. High-difference features can mislead the model and lead to negative transfer, while low-difference features are the key to knowledge transfer. After parallel training RF, GBM, and TL learners in the same hierarchical cross validation framework and fusing them in a “hard voting” manner, the macro indicators of each model on the bearing dataset were compared, as shown in Table 4.

**Figure 12:**
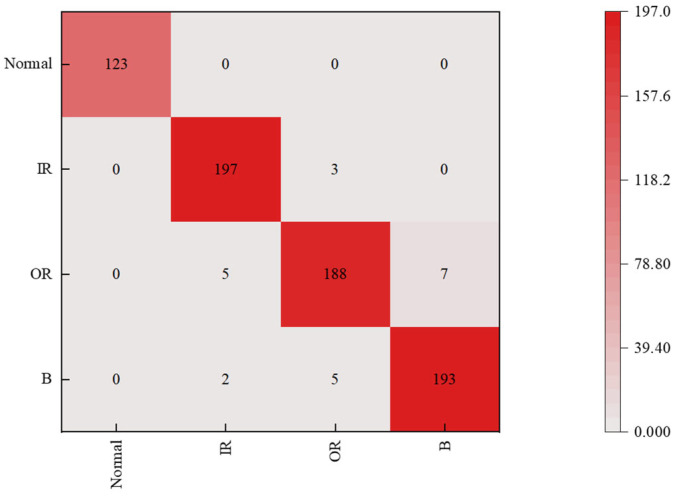
Prediction results of the MLP model.

The experiment showed that TL had the best comprehensive performance: the testing accuracy was 98.63%. The prediction accuracy and classification results of this model are shown in Figure 12.

The comparison results of transfer learning effects are shown in Table 5.

The fault diagnosis results of the target domain file are shown in Table 6.

## 5. Sensitivity Analysis

The application of artificial intelligence in safety-critical fields (such as bearing fault diagnosis) is commonly hindered by insufficient interpretability. “Black-box” model decision mechanisms are opaque and struggle to provide physically meaningful diagnostic support, which impedes their practical deployment. Accordingly, this study constructed a multi-level interpretability analysis framework that links prediction results with the physical mechanisms of faults, enabling technicians to intuitively grasp diagnostic foundations and thereby enhance trust in intelligent methods. Among these, SHAP (Shapley Additive exPlanations) is based on game theory and offers rigorous contribution explanations for machine learning predictions [33]. Its core objective is to quantify the contribution of each input feature *x* to the model’s final prediction value *f* (*x*). The SHAP value originates from the Shapley value in cooperative game theory, with the fundamental idea that a feature’s contribution should be measured by examining the average marginal impact on model output when the feature is included in all possible subsets of features [34]. For a model with *M* features, the Shapley value *ϕ*(*i*) of feature *i* is calculated by the following formula.

(33) $$\phi_{i}=\sum_{S\subseteq M\backslash \left\{i\right\}}\frac{\left|S\right|!(M-\left|S\right|-1)!}{M!}\left[f(S\cup \left\{i\right\})-f(S)\right],$$

where S is a feature subset that does not include the feature i; f(S) represents the model’s predicted value when only using the features in the subset S; $f(S\cup \left\{i\right\})$ is the predicted value after adding the feature i; and the weight term $\left|S\right|!(M-\left|S\right|-1)!/M!$ is used to fairly weigh all possible subset combinations.

The core advantage of the SHAP method lies in its additivity property [35]. For any sample, the sum of SHAP values for all features equals the difference between the sample’s predicted value and the model’s average baseline prediction:

(34) $$f(x)=\phi_{0}+\sum_{i=1}^{M}\phi_{i}.$$

where f(x) is the predicted output of the model on the sample x; $\phi_{0}$ is the baseline value (i.e., the expected prediction of the model when all features are missing, such as the average prediction value of the training set); and $\sum_{i=1}^{M}\phi$*_i_* is the sum of all feature contribution values. This property enables SHAP to provide highly intuitive local explanations: we can clearly see whether each feature “pushes” or “pulls” the predicted result from the baseline value, as well as its degree of influence.

Integrated gradients, introduced by Sundararajan et al. [36], is a prominent technique in the family of attribution methods. It assigns importance scores to input features, which can explain the model’s predictions. Specifically, this method quantifies the contribution of each feature to the model output by calculating the path integral of the gradient relative to the baseline input. Integrated gradients require baseline inputs as reference points, which typically represent the neutral or uninformative state of the model, such as zero vectors or random noise. This method evaluates the importance of each feature by integrating gradients along a straight path from the baseline to the actual input in the input space, as shown in the following formula:

(35) $$Integrated\;Gradients_{i}(x)=(x_{i}-x_{i}^{\prime })\times \int_{\alpha =0}^{1}\frac{\partial F(x^{\prime }+\alpha (x-x^{\prime }))}{\partial x_{i}}d\alpha .$$

In the formula, x represents the model input; $x^{\prime }$ represents the baseline input zero vector (which defaults to zero vector); F is the output function of the model; parameter α serves as the interpolation factors to define the path between the baseline and the actual input; and $\partial F\left(x\right)/\partial x_{i}$ corresponds to the gradient of F(x) of the i-th dimension. The final result assigns an importance score to each input feature, where positive values represent the positive contributions to the model output and negative values represent the negative contributions.

For different categories in the target domain, the extracted features are of significant importance, especially the root mean square, peak factor, and BPFI amplitude, which are particularly important for fault diagnosis.

## 6. Conclusions

The advantage of transfer learning lies in its ability to significantly reduce the data and annotation requirements of the target domain, enhance model training speed and generalization capability, and address cold-start issues in the absence of historical data. This study comprehensively captured the key fault-related information from vibration signals by integrating multi-dimensional features, such as time and frequency domains. By employing transfer learning techniques, it effectively mitigates the modeling difficulties caused by scarce target-domain samples and imbalanced label distributions. The model demonstrates stable recognition performance across various operating conditions, exhibiting strong adaptability and generalization potential. The fine-tuning strategy optimizes the knowledge transfer path from the source domain to the target domain, improving transfer efficiency. Through transfer learning modeling of vibration signal data, the following conclusions were drawn: (1) The transfer learning model achieves a 27.3% increase in test accuracy and a 0.119 rise in average confidence compared to directly applying the source-domain model. (2) The transfer learning model’s test accuracy, F-1 score, and cross validation accuracy significantly outperform those of the GBM and RF models, with a lower standard deviation in test results. (3) Among all features, the root mean square and crest factor exhibited the highest importance, best reflecting equipment fault conditions. However, existing transfer learning methods primarily rely on statistical distribution alignment, facing significant adaptation challenges in cross domain scenarios with substantial distribution disparities, making it difficult to effectively overcome the performance degradation caused by fundamental domain differences. When confronted with overly complex or highly noisy data, the model may overfit, reducing prediction accuracy.

Future research efforts can be directed in the following aspects: first, developing more advanced domain adaptation techniques, such as deep domain adaptation or adversarial training methods, to enhance the model’s robustness against distribution shifts; second, exploring multi-source domain transfer learning frameworks that leverage knowledge from multiple source domains to improve target domain performance; and third, investigating zero-shot or few-shot learning techniques to address extreme scenarios with severely scarce target domain data. Additionally, this model demonstrates excellent generalizability, and its application scope can be further extended to the state monitoring and fault diagnosis of other industrial mechanical systems, such as motors and gears. The proposed transfer learning framework effectively mitigates the challenges arising from equipment heterogeneity, variable operating conditions, and insufficient labeled samples, providing a scalable solution for building intelligent manufacturing and equipment health management systems.

## Figures and Tables

**Table 1:** Evaluation indicators.

	Accuracy	Precision	Recall	F1-Score
Before feature extraction	0.8913	0.8986	0.8913	0.8918
After feature extraction	0.9823	0.9926	0.9913	0.9811
Trend	+9%	+10%	+10%	+9%

**Table 2:** Importance ranking of the time-domain and frequency-domain features.

Feature	Priority Ranking
Time domain_standard deviation	0.099587
Time domain_Root Mean Square(RMS)	0.089320
Time domain_Peak factor	0.087258
Frequency domain_Mean frequency	0.083697
Frequency domain_Frequency center of gravity	0.077123
Time domain_Pulse factor	0.073173
Time domain_kurtosis	0.070065
Frequency domain_Frequency variance	0.065788

**Table 3:** Bearing size parameters of source domain dataset.

Bearing Type	Number of Scrolls *n*	Scroll Bar Diameter *d*	Bearing Diameter *D*
SKF6205 (DE)	9	0.3126 inch	1.537 inch
SKF6203 (FE)	9	0.2656 inch	1.122 inch

**Table 4:** Macro indicators for each model.

Model	Test Accuracy	F1-Score	Cross Validation Accuracy	Standard Deviation
TL	97.63%	0.9631	95.44%	0.0012
GBM	87.26%	0.8953	90.97%	0.0058
RF	80.11%	0.8196	80.66%	0.0295

**Table 5:** Comparison of the transfer learning effects.

Method	Accuracy	Average Confidence Level	Transfer Gain
Source-domain model	70.3%	0.713	-
Transfer learning model	97.6%	0.832	+27.3%

**Table 6:** Target-domain fault diagnosis results.

Failure Type	Number of Files	Proportion (%)	Average Confidence Level
Outer ring fault	10	62.5	0.859
Normal state	4	25.0	0.821
Inner ring fault	1	6.25	0.763
Rolling element failure	1	6.25	0.702
Total	16	100.0	0.832

## Data Availability

The data in this article is sourced from the CWRU dataset (https://csegroups.case.edu/bearingdatacenter/pages/download-data-file, accessed on 24 November 2025). The CWRU dataset is a bearing fault diagnosis dataset provided by Case Western Reserve University, and it is widely used in mechanical fault diagnosis research.

## Abbreviations

The following abbreviations are used in this manuscript:

TL	Transfer Learning
GBM	Gradient Boosting Machine
RF	Random Forest
SHAP	SHapley Additive exPlanations
